# Preliminary Feasibility of Near-Infrared Spectroscopy to Authenticate Grazing in Dairy Goats through Milk and Faeces Analysis

**DOI:** 10.3390/ani13152440

**Published:** 2023-07-28

**Authors:** Pablo Rodríguez-Hernández, Cipriano Díaz-Gaona, Carolina Reyes-Palomo, Santos Sanz-Fernández, Manuel Sánchez-Rodríguez, Vicente Rodríguez-Estévez, Nieves Núñez-Sánchez

**Affiliations:** Department of Animal Production, Faculty of Veterinary Medicine, University of Cordoba, Campus Rabanales, 14071 Cordoba, Spain; pa2digac@uco.es (C.D.-G.); v22repac@uco.es (C.R.-P.); v22safes@uco.es (S.S.-F.); pa1sarom@uco.es (M.S.-R.); pa2nusan@uco.es (N.N.-S.)

**Keywords:** ruminants, grazing authentication, chemometrics, NIRS

## Abstract

**Simple Summary:**

Nowadays, society demands certification and authentication methodologies that are able to clarify the origin of different livestock products. This is considered of paramount importance in order to not only provide accurate information to consumers, but also to protect producers against fraudulent practices. In this context, the aim of this study is to establish a methodology to authenticate the grazing activity of dairy goats. To achieve this, milk and faeces samples were analysed using Near-Infrared Spectroscopy. The good results obtained in discriminant models demonstrated differences in both types of matrices when the two feeding regimes were compared. The development of this methodology could extend its use not only in dairy systems of goats but also in other animal species and systems.

**Abstract:**

Consumers are increasingly prone to request information about the production systems of the food they buy. For this purpose, certification and authentication methodologies are necessary not only to protect the choices of consumers, but also to protect producers and production systems. The objective of this preliminary work was to authenticate the grazing system of dairy goats using Near-Infrared Spectroscopy (NIRS) analyses of milk and faeces of the animals. Spectral information and several mathematical pre-treatments were used for the development of six discriminant models based on different algorithms for milk and faeces samples. Results showed that the NIRS spectra of both types of samples had some differences when the two feeding regimes were compared. Therefore, good discrimination rates were obtained with both strategies (faeces and milk samples), with classification percentages of up to 100% effectiveness. Discrimination of feeding regime and grazing authentication based on NIRS analysis of milk samples and an alternative sample such as faeces is considered as a potential approach for dairy goats and small ruminant production.

## 1. Introduction

Nowadays, consumers are more aware about food quality, authenticity, origin, labelling and ingredients than ever [1]. Regarding dairy products, consumers demand information about animals’ diets, as well as the systems in which they were raised [2,3,4]. Hence, the demand for traditional, regional and organic products, which are perceived as healthy and added-value foods, has increased [5,6]. In this context, denominations of origin, quality certificates and discrimination methodologies are considered of great importance to ensure consumer food safety and choice [7] as well as to obtain profitability for small producers [8]. However, although there are some national and international regulations focused on food labelling and trade, these regulations and their controls are frequently perceived as insufficient [1].

Animal products from grazing systems, where pastures and other natural resources become the animal diet basis (although grains or concentrate feed are also employed), have a more positive image than those from intensive systems [9]. Therefore, products from these systems are often preferred by consumers due to their origin from animals with a more natural feeding regime, since animal diet is considered by consumers today as an essential criterion for determining the quality of products [10,11]. The feeding regime of animals is directly related to food properties and safety, but this connection is often not clear for consumers, who therefore do not have enough information to make a proper choice [12].

Previous studies to authenticate the feeding regime of origin from food products employed direct and indirect analyses such as triacylglycerols species’ evaluation in milk and dairy products [13], the use of nuclear-magnetic-resonance-based metabolomic markers to authenticate beef production systems [14], the evaluation of hippuric acid as a marker of feeding regime in goat milk [15], or the categorization of organic foods through the study of certain biomarkers [16]. These studies require complex methodologies or the use of previously established specific markers, which are not always easily definable [17]. 

Near-Infrared Spectroscopy (NIRS) or NIRS technology is based on the correlation of physico-chemical properties of a product and the absorption of radiation in the infrared region; it is considered a rapid, non-destructive, cheap, and user-friendly methodology that is able to determine a great deal of parameters with high repeatability and reproducibility without the need to set prior classification criteria [18]. Chemometric methods are used afterwards to develop discriminant and predictive models which correlate the spectral data to the quantitative and/or qualitative attributes of the samples [19]. The technological progress both in chemometrics and in NIRS instrumentation has induced its implementation as an analytical tool in the agri-food industry [20]. Different samples such as dairy products [4,10], meat [21,22] or honey [23,24] have been successfully analysed through NIRS technology with different quantitative and qualitative aims, thus highlighting its versatility. NIRS has already been evaluated as an analytical tool to discriminate animal feeding regimes: for example, a high classification success was obtained after NIRs analysis of cheese and milk to study and differentiate ruminants’ diet [8,10]; this is considered as a promising approach for authenticating products from grass-fed animals [25]. However, despite the interest of this application for authentication purposes in the dairy sector, NIRS technology has been mainly used with cow-derived products, and only scarce information about small ruminant products has been found in the literature.

Most of the NIRS studies reporting dairy systems’ authentication usually evaluate final products such as milk or cheese. However, previous works have shown that faeces spectra have sufficient information to quantitatively predict the chemical composition or digestibility of fed diets in different species such as pigs [26], rabbits [27] and ruminants [28,29]. In this sense, Ottavian et al. [30] also addressed the use of faecal NIRS analysis to successfully discriminate dairy cows among two levels of concentrate supplementation. Hence, faeces offer some advantages as an interesting and alternative sample for authentication, due to their ease of collection as well as the information they provide about the individual physiology, including animal feeding [31]. Thus, faeces may also constitute an alternative and good option to discriminate and certify the grazing practice in livestock farming. 

The objective of this present study is to evaluate the preliminary viability of NIRS technology to authenticate grazing of dairy goats and its discrimination from intensive feeding, where concentrate feed constitutes the diet base. For this aim, two different biological samples, milk and faeces, were studied in order to compare the results obtained in the discriminant models.

## 2. Materials and Methods

### 2.1. Animals and Samples

The present study was carried out using a total of 71 individual milk samples and 66 individual faeces samples from Florida goats, an autochthonous breed of southern Spain. The animals included in the study were adult females with an age ranging between two and three years. Samples were collected in two farms in the province of Cordoba: 39 milk and 35 faeces samples belonged to an intensive farm, and the remaining 32 milk and 31 faeces samples belonged to a farm where the goats had an important food input through natural grazing in addition to conventional dairy feeding. Both farms held an M4 brucellosis status (officially brucellosis-free) when the samples were collected. All samples were collected in spring of 2021. The two farms included in the present study were selected due to their similarity in terms of productive conditions, animal health, as well as genetics. In addition, the help and collaboration of farmers were also considered within the selection criteria. Milk and faeces samples were analysed in order to perform a direct authentication using the final product on one hand (milk) and, on the other hand, to evaluate an alternative matrix (faeces).

Both farms included in the study belonged to the National Breeders Association of Florida goats (ACRIFLOR), where technicians ensure equal productive conditions but consider different feeding regimes as the differential factor. While the animals at the intensive farm were permanently stabled in different pens, the goats at the second farm grazed in different paddocks ranging from 16 to 32 ha, depending on the number of animals per batch. Those animals grazed during the time between milkings, which were conducted in the early morning (7–8 a.m.) and early afternoon (4–5 p.m.). After the second milking, the goats were stabled indoors. Dehesa with Mediterranean scrubs and olive groves were the main vegetation in the grazing paddocks.

Therefore, the diet of animals was based on concentrate feed in the intensive farm and a combination of concentrate feed and natural resources obtained while grazing in the second farm. The commercial feed used in both farms was based on cereals and legumes, with an average nutritional composition of 16.6–18% of crude protein, 6.62–7.3% of crude fibre, 7.98% of ash, 3.17–3.57% of ether extract, 0.98–1.05% of calcium, 0.43% of phosphorous and 0.22–0.47% of sodium. The amount of commercial feed given to the goats in both farms was approximately 2 kg feed/animal per day (1.74 kg of dry matter). The rest of the daily intake was covered with gramineous and legume hay in the intensive farm and with pastures and different grazing resources in the second farm. In addition, in both farms, the goats were offered cereal straw ad libitum. Considering an average daily intake of 2.5 kg of dry matter for an adult dairy goat in production [32], the intake obtained from grazing was approximately 25–30% of the total daily intake. Animals were on the above-described feeding regime for at least one month before sampling.

Individual milk and faeces samples were collected during milking to avoid unnecessary manipulations and stress. Milk samples were collected after teat cleaning and before teat cup attachment. Faeces samples were collected per rectum in order to prevent sample degradation or contamination. The animals without enough faeces in their rectum were removed from the experiment to avoid stress during milking. All samples were stored and transported to the Animal Production Laboratory of the University of Cordoba and were frozen at −18 °C until their processing and NIRS analysis.

### 2.2. Sample Processing and Near-Infrared Spectra Collection

Faecal samples were oven-dried at 60 °C for 24 h, and then, they were milled to pass a 1 mm sieve. The DESIR (dry-extract system for infrared) methodology adapted for milk [33] was used for the NIRS analysis of the samples as follows: milk samples were unfrozen by immersion in a water bath at 40 °C, mixed gently to achieve uniform dispersion of their components, and then left to cool at room temperature. A glass microfibre filter (Millipore, AP4004705, Merch, Madrid, Spain) per sample was impregnated with milk and then oven-dried at 40 °C for 24 h. 

A FOSS-NIRSystems 5000 scanning monochromator (FOSS-NIRSystems, Silver Spring, MD, USA) equipped with a transport module was used for sample analysis. Spectral absorbance values were recorded in reflectance mode from 1100 to 2498 nanometers (nm) every 2 nm, as log 1/R, where R is the sample reflectance. Each spectrum was time-averaged from 32 scans and compared with the 16 measurements of a ceramic reference. Spectra of the oven-dried milk and faeces samples were acquired using a small ring cup, thus obtaining one spectrum per sample.

### 2.3. Data Processing and Discriminant Models Development

The WinISI IV software package (version 4.8, Foss, Hillerød, Denmark), MATLAB^®^ software (The Mathworks Inc., Natick, MA, USA, 2007) and its plugin PLS Toolbox (Eigenvector Research, Inc., Manson, WA, USA) were used for the chemometric treatment of the data. Prior to the development of the discriminant models, a principal component analysis (PCA) was carried out to observe the structure of the spectral population and to detect possible outlier samples. PCA is an exploratory method to reduce the dimensionality of data matrices that retains as much variability of the spectral data as possible, thus allowing detection of anomalous or outlier spectra. Samples whose Mahalanobis distance or spectral distance to the centre of the spectral population was greater than 3 were considered anomalous and were eliminated. Discriminant models were developed separately for the milk and faecal samples with outlier-free sample sets. A similar procedure was followed in both cases, as described below:

Discriminant models used exclusively spectral information for their classification in groups, and no chemical lab information was required. As the WINISI IV program requires that the samples belonging to each group to be included in separate files, “grazing” and “intensive” files were created for both the milk and the faeces sample sets, containing the samples from the animals belonging to the grazing and intensive production systems. Furthermore, to validate the discriminant models, each group (grazing and intensive) was randomly divided into calibration and external validation sets with 80% (n = 111) and 20% (n = 26) of the samples, respectively. Samples from the validation set were used as “blind” samples to evaluate the effectiveness of the model calibrated. 

Several mathematical pre-treatments were used in this work. Firstly, spectral derivatives were employed to eliminate additive and multiplicative effects in the spectrum [34]. These were named using four digits: the first digit is related to the order of the derivative (1 = 1st derivative; 2 = 2nd derivative); the second digit refers to the size of the derivation segment (interval expressed in nm, where the calculation of the derivative is performed); and the third and fourth digits refer to the treatment of the smoothing segments (intervals, expressed in nm, used for the smoothing calculation). Spectral derivatives 1, 5, 1, and 1 and 2, 5, 1, and 1 were used in this work, following the recommendations of the WINISI IV chemometric software for discriminant models. In combination with derivatives, the standard normal variate and detrending algorithms (SNV + DT) were used to correct the scatter effect [35]; albeit, models were also performed without scatter correction. Thus, four discriminant models were obtained for each algorithm in the spectral region of 1100–2500 nm: 1, 5, 1, 1 and 2, 5, 5, 1, both with SNV + DT and without scatter correction.

All 6 discriminant algorithms available in the WINISI IV version 4.8 chemometric software were used for the development of the discriminant models. Each algorithm provided a different mathematical approach to the discrimination. The foundations for each discriminant algorithm used in this work is described below:

PLS2: this algorithm creates a dummy variable to denote group membership, assigning a value of 2 if the spectrum belongs to one group, and a value of 1 to the other samples. A 2-block PLS regression is performed with the dummy variable to create calibrations that predict values for group parameters. The value of 1.5 is established as the limit value between both groups, so that samples with a predicted value above 1.5 belong to the group, and those below 1.5 do not belong to the group.

Correlation: in this algorithm, a mean spectrum is calculated for each group. Each sample is assigned to the group with the highest correlation and, thus, a higher value of the classification variable.

Maximum distance: the algorithm calculates the mean spectrum of each group and calculates the distance of each sample to the mean spectra at every datapoint. The sample is assigned to the group with the lowest maximum distance, i.e., the one with the lowest value of the classification variable.

Mahalanobis distance: this algorithm calculates the centre of each group and the Mahalanobis distance (H) of each sample to that centre. Samples are assigned to the closest group, i.e., the one in which they have the lowest H value.

X-residuals: this algorithm calculates the model spectrum for each group and reconstructs each spectrum to make it similar to the model one. The difference between the original spectrum and the reconstructed one is called the X-residual. The samples are assigned to the group with the lowest X-residual value.

Maximum X-residuals: this discriminant algorithm combines all the previous discriminant procedures, providing a maximum X-residual value for each sample. Samples are assigned to the group with the lowest maximum X-residual value.

During discriminant model development, the algorithms assigned the samples to one of the groups: if it was assigned to its origin group, it was considered as correct or correct classification; if it was classified in a group other than the one of origin, it constituted a failure. The best algorithm to discriminate milk and faeces groups was selected according to the highest percentage of correctly classified samples in calibration and external validation.

## 3. Results

### 3.1. NIRS Spectra Evaluation

The individual evaluation of each spectrum obtained after NIRS analysis revealed no anomalies for milk samples. However, after faeces spectra evaluation, the spectrum of one sample belonging to the grazing group was removed from the set due to an anormal absorbance. This finding may be related to a possible failure during sample preparation or during the subsequent analysis. Therefore, the final number of samples employed for discriminant model development were 71 in the case of milk samples and 66 faeces samples. 

**Figure 1:**
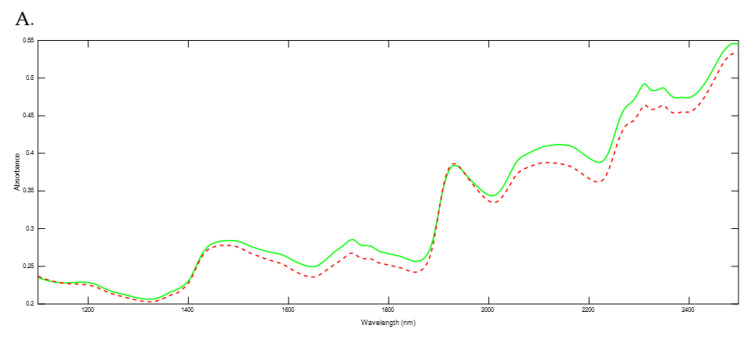

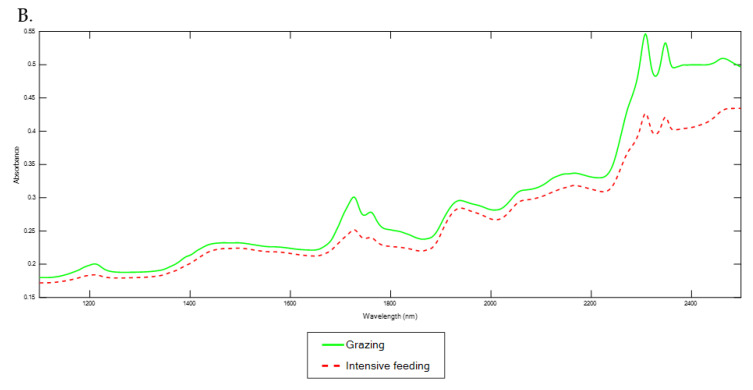
Mean Near Infrared Spectroscopy (NIRS) spectra for faeces (**A**) and milk (**B**) samples considering the two feeding strategies evaluated.

The mean spectra of both feeding strategies were compared for milk and faeces samples; the comparison is available in Figure 1. Different absorption bands can be distinguished along the spectra: two slight bands around 1450 and 1940 nm are mostly characteristic of moisture absorption [27,34]. Bands at 1210, 1726, 1760, 2308 and 2348 nm correspond to fatty acids and fat content [36]; bands at 1516, 2056, 2174, and 2468 nm correspond to protein absorption [37]; and bands at 1922, 2078–2110, 2268 and 2420 nm in the faecal spectra correspond to fibre content [38].

In general, a greater absorbance was obtained for the grazing group in comparison with the intensive feeding group, a difference which can be noticed in both faeces (Figure 1A) and milk samples (Figure 1B). Although this difference was present over almost the entire mean spectra, there were some areas where it was more pronounced: the spectra of both sample sets had a higher absorbance for the grazing group in bands at 1650–1750 nm and 2250–2350 nm, which correspond, as above-mentioned, to the fat content, underlining the possible influence of this component for the discrimination between the two feeding regimes evaluated.

### 3.2. PCA Using NIRS Spectra of Faeces and Milk Samples

**Figure 2:**
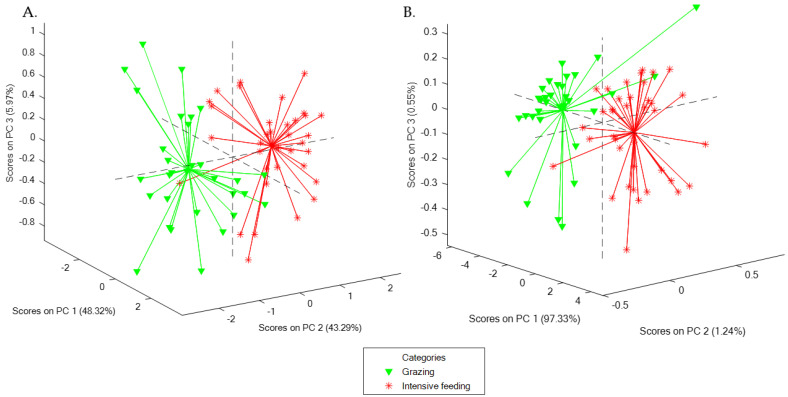
Score plots of the two Principal Component Analysis (PCA) performed: (**A**) PCA using NIRS spectra of faeces samples; (**B**) PCA using NIRS spectra of milk samples.

In parallel with spectra evaluation, PCA did not highlight any outliers among samples evaluated with NIRS technology; hence, all the samples were employed for the chemometric models except the sample excluded during the previous evaluation of NIRS spectra. PCA allows for exploring the distribution as well as the tendencies and tentative differences between the samples analysed. In this sense, some interesting findings may be addressed from both PCA obtained with faeces and milk samples (Figure 2). First, although PCA is not a discriminant model, a slight separation between grazing and intensive feeding groups is appreciated in the score plot of both matrices. A different spatial location was observed for each farm or feeding group, which seemed to concentrate in different areas of the graphic. Furthermore, according to the size of the point clouds, a different distribution of samples was observed between the two feeding strategies compared: while the samples of the intensive feeding group were concentrated around a specific area of the PCA graphic, grazing goats’ samples showed a higher dispersion (larger point cloud size).

### 3.3. Discriminant Models

The tentative and initial differences noticed between the two feeding strategies during the PCA evaluation were confirmed afterwards with the discriminant model development.

As stated previously, discriminant models were performed separately for faeces and milk samples using exclusively the spectral information. In each population, 80% of the samples was used to develop the models, and the remaining 20% was used to validate them. Four combinations of mathematical treatments in the NIRS spectral region of 1100–2500 nm were used in each of the six discriminant algorithms: PLS2, correlation, maximum distance, Mahalanobis distance, X-residuals and maximum X-residuals. The results for milk and faeces samples are presented separately to ease comprehension.

#### 3.3.1. Faeces Samples

The results of the faeces discriminant models are shown in Table 1. Data related to confusion matrices are available in Supplementary Table S1. In general, high classification percentages were reached in both the calibration and the external validation of models, although some differences were found among the algorithms and the mathematical treatments used.

The PLS2 algorithm reached an excellent calibration, with 100% classification success with all the mathematical treatments except 2, 5, 1, 1 and SNV + DT, which obtained 96.4% calibration success. Validation results obtained with PLS2 were also high, reaching 100% success with two of the mathematical treatments and 81.8% and 90.9% in the two remaining ones. In contrast, the results obtained with the correlation algorithm were lower, with calibration and validation success ranging between 81.8% and 90.9%. Models using the maximum distance algorithm showed high calibration classification (94.5–96.4%), although validation rates were significantly lower (54.5–81.8%). The Mahalanobis distance algorithm obtained an acceptable calibration success (87.3–89.1%) but variable validation success (54.5–100%). The results with the X-residuals algorithm achieved 100% correct classification both in calibration and validation, except for the case of 1, 5, 1, 1 and no scatter mathematical treatment, which reached 90.9% validation success. Lastly, the success obtained by the maximum X-residuals algorithm ranged between 96.4% and 100% in calibration and between 90.9% and 100% in validation.

Overall, the best results were obtained with the PLS2, X-residuals and maximum X-residuals algorithms, which all reached 100% correct classification in both calibration and external validation. Furthermore, a better performance of the discriminant models (in both calibration and validation) was observed when using the second derivative for the mathematical treatment in comparison with the first derivative, either with SNV + DT or with no scatter correction. In contrast, there seemed to be no clear relationship between SNV + DT or no scatter effect correction and the obtention of higher classification results. Furthermore, a significative finding was detected regarding the validation results, since every misclassified sample belonged to the grazing group in most of the algorithms’ combinations (Table S1).

#### 3.3.2. Milk Samples

The calibration and external validation results from the discriminant models performed using NIRS spectra of milk samples are also summarised in Table 1. Data related to confusion matrices are available in Supplementary Table S2. In general, calibration results showed slightly lower classification rates with milk NIRS spectra in comparison with the faeces data; higher variability in the accuracy was observed in validation results, depending on the algorithm and the mathematical treatment used.

The PLS2 algorithm achieved a variable classification success in calibration, ranging between 78.6% and 98.2%, as well as in validation, ranging from 73.3% to 100%. The correlation algorithm achieved a good calibration success (85.7–92.9%) and the highest results in model validation using milk NIRS spectra (93.3–100%). The models performed using the maximum distance algorithm also obtained good success in both calibration (78.6–92.9%) and validation, where a higher classification percentage was achieved by employing the first derivative (100%) in comparison with the second derivative (80%). The Mahalanobis distance algorithm showed similar classification successes in calibration (76.8–92.9%) and validation (73.3–93.3%). The X-residuals algorithm achieved a calibration success ranging between 89.3% and 98.2% and a validation success of 73.3–100%. Discriminant models using the maximum X-residuals algorithm obtained the highest percentages of correct classification in calibration (96.4–100%), while their validation success was slightly lower (80–93.3%). 

The highest effectiveness was obtained with the maximum distance and X-residuals algorithms, which achieved a success of 98.2% and 100% in calibration and validation, respectively. None of the discriminant models achieved 100% correct classification in both calibration and validation. In general, no clear connection between any mathematical treatment (no scatter correction, SNV + DT, first or second derivative) and the obtention of higher classification rates was detected in milk samples.

Similarly to faeces samples, misclassified samples found in the external validation of milk discriminant models tended to belong to the grazing group (Table S2), although the separation between the two feeding groups in the score plot was not as clear as in the faeces models (Figure 2). In this sense, while an acceptable separation between the two feeding strategies was found for faeces (Figure 2A), samples from both diets were closer for milk samples (Figure 2B): this can be seen by looking at the overlapping samples in the score plot, which are higher in the case of milk, with samples from both groups almost overlapped in the graphic (Figure 2B). Therefore, the influence of diet seems to be higher in faeces than in milk samples, at least at a spectral level.

## 4. Discussion

### 4.1. NIRS Spectra Evaluation

The differences related to fat composition and its possible influence on the discrimination between the two feeding regimes noticed during NIRS spectra evaluation of both matrices are consistent with previous results. Capuano et al. [16] highlighted that the authentication of cow fresh herbage feeding was largely related to the wavelengths that are representative of fat content in milk. In fact, the feeding strategy used for cows has been considered to be the most powerful factor in milk fat composition modification [25,39]. All of this is in line with the influence of pasture feeding—and therefore grazing—on the fat composition of ruminant products, which has been underlined before [25]. The obtention of a higher fat content for the grazing strategy in the present study could be due to a direct transfer from the ration to the samples or due to a transformation produced by the individual metabolism or the rumen microbes [25].

### 4.2. PCA Using NIRS Spectra of Faeces and Milk Samples

With respect to the PCA of faeces and milk data, the differences highlighted between the two feeding groups are consistent considering the two strategies followed for the feeding of the animals. Goats included in the intensive feeding group were fed exclusively with concentrate feed and hay, which would be translated into homogeneous NIRS spectra of both milk and faecal samples. On the other hand, animals of the grazing group also had an important nutritional contribution coming from different natural resources while grazing, which was estimated to be approximately 20–30% of the daily dry matter intake. Thus, the huge diversity of resources exploited, as well as the differences in terms of animal preferences along grazing [40], resulted in heterogeneous spectra in this group. This higher variability provided larger point clouds, as samples were more dispersed than the samples of the intensive feeding group, which tended to be more concentrated because of the homogeneous diet.

Grazing in this dairy goat breed has been deeply studied before, highlighting an important heterogeneity and variability of natural resources included in the diet [41,42,43]. In fact, results about the grazing behaviour of different ruminant species previously published by Celaya et al. [44] pointed out goats as a species which graze for a longer time and utilise significantly more vegetation [44]. Similar findings have been underlined before with other species such as the Iberian pig, where some of the analytical results obtained after the comparison of grazing and intensive diets were related to the diet heterogeneity and the individual preferences of the animals in the field [45]. Therefore, the tentative differences observed between PCA results of milk and faeces spectra of the two diets are associated to animal grazing behaviour and diet.

### 4.3. Discriminant Models

#### 4.3.1. Faeces Samples

The external validation results obtained in the discriminant models using faeces spectra, where misclassified samples tended to belong to the grazing group, may be explained by attending to the above-mentioned discussion in Section 4.2 and may be directly related to the diet of animals. In this sense, while intensive feeding would result in homogeneous NIRS spectra of faeces because of the lack of diet variability, grazing goats browse diverse natural resources while grazing, hence obtaining heterogeneous spectra. This fact has already been highlighted before attending to the dispersion of points in the score plots of both groups (Figure 2). Moreover, and as already mentioned, these animals could also express their individual preferences when grazing, contributing to a higher variability of NIRS spectra. Therefore, the inclusion of a higher number of samples that cover as much variability as possible regarding the animal behaviour and the vegetation and pasture diversity would be of paramount importance in order to improve the robustness of the discriminant models.

Despite the scarce literature about grazing authentication in small ruminants, there are some interesting studies employing NIRS and faeces samples in this sense. Landau et al. [46] carried out a review work where an evaluation about the contribution of NIRS technology for the prediction of feed characteristics as well as the botanical composition of grazing diets through faeces analysis was conducted. Dixon and Coates [28] reviewed the potential of NIRS to provide useful information about the nutrition and physiology of herbivores through faeces analysis, including grazing goats. However, these studies were mainly focused on NIRS usefulness to predict and estimate some diet constituents and attributes, without attending to the comparison and discrimination between different feeding strategies. 

In this sense, in addition to the interesting determinations and estimations of diet parameters, nowadays, there is a challenge based on the development of rapid, easy and low-cost methodologies able to authenticate products with attributes appreciated by consumers, such as grazing products [47]. Thus, considering that faeces constitute a biological sample which provides useful information about the physiology, feeding and ecology of the animals [28], and also according to the present results, faecal NIRS analysis may constitute a potential strategy to value grazing systems and products. Moreover, faeces also offer advantages related to being easy and non-invasive to collect; this advantage is considered even more important in grazing and extensive systems, where animals are not always reachable or even observed, which hampering their handling [28]. In this sense, faeces could be collected after spontaneous defaecation during certification or inspection visits. It would not require any animal restraint or immobilisation, as currently conducted in the milking parlour for milk sampling.

Overall, NIRS analysis of faeces could be considered as a potential strategy which allows the indirect certification of the resulting products from grazing animals, such as milk, cheese or meat. The combination of an alternative sample (faeces) and an innovative methodology (NIRS) could contribute to the development of authentication methods of added-value products susceptible to fraud, satisfying the demands of both farmers and consumers, who advocate for the development of analytical methodologies that go beyond on-farm inspection, self-inspections, visual determinations, or audits by independent companies [25].

#### 4.3.2. Milk Samples

The satisfactory results obtained with milk samples after NIRS analysis are consistent with previous findings in the literature, where a clear influence of animal feeding to milk composition has been highlighted [48]. In this sense, the inclusion of grazing in dairy goat production systems would result in milk spectral differences and, consequently, in a successful discrimination with regard to a typical intensive diet (feed and hay). In fact, with respect to that background, many studies have already been conducted for the authentication of animal feeding regimes and grass-fed diets using dairy products; this topic was recently reviewed by Prache et al. [25]. Similar classification rates have been obtained in comparison with the present results: for example, a 100% success was achieved for the discrimination of cows mainly fed with pasture using both milk [49] and cheese samples [50], although complex analyses of isotopic, molecular and lipids markers were employed. Among the methodologies and analytical tools employed for grass-fed product authentication, Prache et al. [25] highlighted NIRS as one of the most promising options due to its advantages (rapidity, low cost and accuracy), which allows for possible routine use of this method.

With respect to the spectral differences induced in milk by feeding regimes (and specifically by grazing), several authors have previously highlighted that fat profiles could be responsible for those differences. For example, Engel et al. [49] showed that, in comparison with other milk components, fatty acids had the best discriminatory power to differentiate milk from cows mainly fed with grass or with maize silage. Also, Coppa et al. [51] found that milk fatty acids were useful to predict diet composition and authenticate feeding systems in cows. In fact, this fat profile in milk would be not only useful to discriminate between different diets, but also between similar ones; several studies which show that the fatty acids even allow discrimination between cultivated and natural pastures exist [52]. These findings are consistent with the results obtained in the present study with milk samples, where the main differences between the two feeding strategies were observed around the fat absorption bands (Figure 1). Therefore, the successful rates obtained for diet discrimination with milk spectra could be partly related to the fat profile. 

Finally, the findings described here have a practical significance because the methodologies used can effectively distinguish between intensive and grazing systems and products, a demand that existed before. This is notable as it benefits extensive producers, allowing clear product differentiation for marketing and consumer trust. Consumers are willing to pay more for authentic products and for products from grazing-based systems, improving profitability for these minority productions, a major challenge nowadays. However, despite the interesting results and the high discrimination rates obtained, the research presented here is considered preliminary, and some limitations related to the number of samples have been identified. Two farms were evaluated in the present study, albeit comparable conditions were ensured within the productive situation of the animals. Therefore, further studies including a greater number of samples, animals and farms are required to obtain more reliable conclusions in the future; this would allow the inclusion of a higher variability, especially regarding pastures and natural resources grazed, and the improvement of model robustness. These aspects are considered of paramount importance for the possible implementation of the effective feeding regime discrimination that was reached in the present study to be available on a large scale. 

## 5. Conclusions

The results of this preliminary study highlight the potential of NIRS technology to discriminate feeding regimes and, therefore, to authenticate grazing in dairy goats. Furthermore, the discrimination was achieved employing not only milk, the most commonly studied sample, but also faeces, which are considered an alternative sample. Several advantages may be addressed to this spectral fingerprint method based on sample optical properties: it is fast, chemical-free and zero-waste, and has the possibility of industrial implementation. The discriminant models performed in this study offer a good rate of success in sample classification for both types of matrices, achieving 100% in calibration and external validation. Therefore, faeces and milk NIRS spectra seem to contain enough information to discriminate and authenticate grazing in organic or grass-fed certified dairy goats. However, additional research is required to extend the potential application evaluated here to the productive sector.

## Figures and Tables

**Table 1:** Calibration and external validation results for the six algorithms performed using Near Infrared Spectroscopy (NIRS) spectra of milk and faeces samples.

Algorithm	Mathematical Treatment	Classification Success			
		Faeces Samples		Milk Samples	
		Model Calibration	Model External Validation	Model Calibration	Model External Validation
PLS2	1, 5, 1, 1 no scatter	100%	81.8%	78.6%	100%
	2, 5, 1, 1 no scatter	100%	100%	94.6%	100%
	1, 5, 1, 1 SNV + DT	100%	100%	98.2%	73.3%
	2, 5, 1, 1 SNV + DT	96.4%	90.9%	80.4%	73.3%
Correlation	1, 5, 1, 1 no scatter	87.3%	90.9%	87.5%	93.3%
	2, 5, 1, 1 no scatter	90.9%	81.8%	92.9%	100%
	1, 5, 1, 1 SNV + DT	87.3%	90.9%	85.7%	93.3%
	2, 5, 1, 1 SNV + DT	90.9%	81.8%	87.5%	100%
Maximum distance	1, 5, 1, 1 no scatter	94.5%	81.8%	94.6%	100%
	2, 5, 1, 1 no scatter	96.4%	81.8%	78.6%	80%
	1, 5, 1, 1 SNV + DT	96.4%	72.7%	98.2%	100%
	2, 5, 1, 1 SNV + DT	94.5%	54.5%	94.6%	80%
Mahalanobis distance	1, 5, 1, 1 no scatter	89.1%	90.9%	87.5%	80%
	2, 5, 1, 1 no scatter	89.1%	90.9%	76.8%	73.3%
	1, 5, 1, 1 SNV + DT	87.3%	100%	92.9%	93.3%
	2, 5, 1, 1 SNV + DT	87.3%	54.5%	85.7%	73.3%
X-residuals	1, 5, 1, 1 no scatter	100%	90.9%	92.9%	73.3%
	2, 5, 1, 1 no scatter	100%	100%	89.3%	100%
	1, 5, 1, 1 SNV + DT	100%	100%	98.2%	100%
	2, 5, 1, 1 SNV + DT	100%	100%	98.2%	93.3%
Maximum X-residuals	1, 5, 1, 1 no scatter	96.4%	90.9%	100%	86.7%
	2, 5, 1, 1 no scatter	98.2%	90.9%	96.4%	93.3%
	1, 5, 1, 1 SNV + DT	96.4%	100%	100%	93.3%
	2, 5, 1, 1 SNV + DT	100%	100%	98.2%	80%

No scatter: no scatter effect correction performed; SNV + DT: standard normal variate and detrending transformation.

## Data Availability

These data are not deposited in an official repository. The data supporting the findings of this study are available upon request from the author Pablo Rodríguez-Hernández (v22rohep@uco.es) and with permission from farmers included in the study.

## Supplementary Materials

The following supporting information can be downloaded at https://www.mdpi.com/article/10.3390/ani13152440/s1, Table S1: Confusion matrices obtained in calibration and external validation for the six algorithms performed using NIRS spectra of faeces samples; Table S2:Confusion matrices obtained in calibration and external validation for the six algorithms performed using NIRS spectra of milk samples..
