# Supplementary material for: Preliminary Feasibility of Near-Infrared Spectroscopy to Authenticate Grazing in Dairy Goats through Milk and Faeces Analysis
Source: Animals (Basel). 2023 Jul 28;13(15):2440. doi: 10.3390/ani13152440 (PMC10417735; doi:10.3390/ani13152440)
Supplement: Supplementary file 1 [file animals-13-02440-s001.zip › animals-2496485-supplementary.pdf]

Table S1. Confusion matrices obtained in calibration and external validation for the six algorithms performed using NIRS spectra of faeces samples.

Algorithm	Mathematical treatment	Model calibration					Model validation				
		Groups		Model prediction: Grazing	Model prediction: Intensive feeding	Classification %	Groups		Model prediction: Grazing	Model prediction: Intensive feeding	Classification %
PLS2	1,5,1,1 no scatter	Real classification	Grazing	26	0	100%	Real classification	Grazing	3	2	81.8%
			Intensive feeding	0	29			Intensive feeding	0	6	
	2,5,1,1 no scatter		Grazing	26	0	100%		Grazing	5	0	100%
			Intensive feeding	0	29			Intensive feeding	0	6	
	1,5,1,1 SNV+DT		Grazing	26	0	100%		Grazing	5	0	100%
			Intensive feeding	0	29			Intensive feeding	0	6	
	2,5,1,1 SNV+DT		Grazing	25	1	96.4%		Grazing	4	1	90.9%
			Intensive feeding	1	28			Intensive feeding	0	6	
		Groups		Model prediction: Grazing	Model prediction: Intensive feeding	Classification %	Groups		Model prediction: Grazing	Model prediction: Intensive feeding	Classification %
Correlation	1,5,1,1 no scatter	Real classification	Grazing	21	5	87.3%	Real classification	Grazing	4	1	90.9%
			Intensive feeding	2	27			Intensive feeding	0	6	
	2,5,1,1 no scatter		Grazing	24	2	90.9%		Grazing	3	2	81.8%
			Intensive feeding	3	26			Intensive feeding	0	6	
	1,5,1,1 SNV+DT		Grazing	21	5	87.3%		Grazing	4	1	90.9%
			Intensive feeding	2	27			Intensive feeding	0	6	

	2,5,1,1 SNV+DT		Grazing	24	2	90.9%		Grazing	3	2	81.8%
			Intensive feeding	3	26			Intensive feeding	0	6	
			**Groups**	**Model prediction**		**Classification %**		**Groups**	**Model prediction**		**Classification %**
				Grazing	Intensive feeding				Grazing	Intensive feeding	
Maximum distance	1,5,1,1 no scatter	Real classification	Grazing	25	1	94.5%	Real classification	Grazing	3	2	81.8%
			Intensive feeding	2	27			Intensive feeding	0	6	
	2,5,1,1 no scatter		Grazing	26	0	96.4%		Grazing	3	2	81.8%
			Intensive feeding	2	27			Intensive feeding	0	6	
	1,5,1,1 SNV+DT		Grazing	26	0	96.4%		Grazing	2	3	72.7%
			Intensive feeding	2	27			Intensive feeding	0	6	
	2,5,1,1 SNV+DT		Grazing	25	1	94.5%		Grazing	1	4	54.5%
			Intensive feeding	2	27			Intensive feeding	1	5	
			**Groups**	**Model prediction**		**Classification %**		**Groups**	**Model prediction**		**Classification %**
				Grazing	Intensive feeding				Grazing	Intensive feeding	
Mahalanobis distance	1,5,1,1 no scatter	Real classification	Grazing	26	0	89.1%	Real classification	Grazing	4	1	90.9%
			Intensive feeding	6	23			Intensive feeding	0	6	
	2,5,1,1 no scatter		Grazing	25	1	89.1%		Grazing	4	1	90.9%
			Intensive feeding	5	24			Intensive feeding	0	6	

	1,5,1,1 SNV+DT		Grazing	26	0	87.3%		Grazing	5	0	100%
			Intensive feeding	7	22			Intensive feeding	0	6	
	2,5,1,1 SNV+DT		Grazing	25	1	87.3%		Grazing	1	4	54.5%
			Intensive feeding	6	23			Intensive feeding	1	5	
X-residuals			**Groups**	**Model prediction**		**Classification %**		**Groups**	**Model prediction**		**Classification %**
				Grazing	Intensive feeding				Grazing	Intensive feeding	
	1,5,1,1 no scatter	Real classification	Grazing	26	0	100%	Real classification	Grazing	4	1	90.9%
			Intensive feeding	0	29			Intensive feeding	0	6	
	2,5,1,1 no scatter		Grazing	26	0	100%		Grazing	5	0	100%
			Intensive feeding	0	29			Intensive feeding	0	6	
	1,5,1,1 SNV+DT		Grazing	26	0	100%		Grazing	5	0	100%
			Intensive feeding	0	29			Intensive feeding	0	6	
	2,5,1,1 SNV+DT		Grazing	26	0	100%		Grazing	5	0	100%
			Intensive feeding	0	29			Intensive feeding	0	6	
Maximum X-residuals			**Groups**	**Model prediction**		**Classification %**		**Groups**	**Model prediction**		**Classification %**
				Grazing	Intensive feeding				Grazing	Intensive feeding	
	1,5,1,1 no scatter	Real classification	Grazing	25	1	96.4%	Real classification	Grazing	4	1	90.9%
			Intensive feeding	1	28			Intensive feeding	0	6	

2,5,1,1 no scatter	Grazing	26	0	98.2%	Grazing	4	1	90.9%
	Intensive feeding	1	28		Intensive feeding	0	6	
1,5,1,1 SNV+DT	Grazing	25	1	96.4%	Grazing	5	0	100%
	Intensive feeding	1	28		Intensive feeding	0	6	
2,5,1,1 SNV+DT	Grazing	26	0	100%	Grazing	5	0	100%
	Intensive feeding	0	29		Intensive feeding	0	6	

No scatter: no scatter effect correction performed; SNV + DT: Standard Normal Variate and De-trending transformation.

Table 2. Confusion matrices obtained in calibration and external validation for the six algorithms performed using NIRS spectra of milk samples.

Algorithm	Mathematical treatment	Model calibration					Model validation				
		Groups		Model prediction: Grazing	Model prediction: Intensive feeding	Classification %	Groups		Model prediction: Grazing	Model prediction: Intensive feeding	Classification %
PLS2	1,5,1,1 no scatter	Real classification	Grazing	18	7	78.6%	Real classification	Grazing	7	0	100%
			Intensive feeding	5	26			Intensive feeding	0	8	
	2,5,1,1 no scatter		Grazing	22	3	94.6%		Grazing	7	0	100%
			Intensive feeding	0	31			Intensive feeding	0	8	
	1,5,1,1 SNV+DT		Grazing	24	1	98.2%		Grazing	4	3	73.3%
			Intensive feeding	0	31			Intensive feeding	1	7	
	2,5,1,1 SNV+DT		Grazing	18	7	80.4%		Grazing	4	3	73.3%
			Intensive feeding	4	27			Intensive feeding	1	7	
		Groups		Model prediction: Grazing	Model prediction: Intensive feeding	Classification %	Groups		Model prediction: Grazing	Model prediction: Intensive feeding	Classification %
Correlation	1,5,1,1 no scatter	Real classification	Grazing	21	4	87.5%	Real classification	Grazing	6	1	93.3%
			Intensive feeding	3	28			Intensive feeding	0	8	
	2,5,1,1 no scatter		Grazing	22	3	92.9%		Grazing	7	0	100%
			Intensive feeding	1	30			Intensive feeding	0	8	
	1,5,1,1 SNV+DT		Grazing	22	3	85.7%		Grazing	6	1	93.3%
			Intensive feeding	5	26			Intensive feeding	0	8	

	2,5,1,1 SNV+DT		Grazing	22	3	87.5%		Grazing	7	0	100%
			Intensive feeding	4	27			Intensive feeding	0	8	
Maximum distance		**Groups**		**Model prediction**		**Classification %**	**Groups**		**Model prediction**		**Classification %**
				Grazing	Intensive feeding				Grazing	Intensive feeding	
	1,5,1,1 no scatter	Real classification	Grazing	24	1	94.6%	Real classification	Grazing	7	0	100%
			Intensive feeding	2	29			Intensive feeding	0	8	
	2,5,1,1 no scatter		Grazing	23	2	78.6%		Grazing	7	0	80%
			Intensive feeding	10	21			Intensive feeding	3	5	
	1,5,1,1 SNV+DT		Grazing	24	1	98.2%		Grazing	7	0	100%
			Intensive feeding	0	31			Intensive feeding	0	8	
	2,5,1,1 SNV+DT		Grazing	24	1	94.6%		Grazing	6	1	80%
			Intensive feeding	2	29			Intensive feeding	2	6	
Mahalanobis distance		**Groups**		**Model prediction**		**Classification %**	**Groups**		**Model prediction**		**Classification %**
				Grazing	Intensive feeding				Grazing	Intensive feeding	
	1,5,1,1 no scatter	Real classification	Grazing	25	0	87.5%	Real classification	Grazing	7	0	80%
			Intensive feeding	7	24			Intensive feeding	3	5	
	2,5,1,1 no scatter		Grazing	23	2	76.8%		Grazing	7	0	73.3%
			Intensive feeding	11	20			Intensive feeding	4	4	
	1,5,1,1 SNV+DT		Grazing	25	0	92.9%		Grazing	7	0	93.3%

			Intensive feeding	4	27			Intensive feeding	1	7	
	2,5,1,1 SNV+DT		Grazing	24	1	85.7%		Grazing	7	0	73.3%
			Intensive feeding	7	24			Intensive feeding	4	4	
		**Groups**		**Model prediction**		**Classification %**	**Groups**		**Model prediction**		**Classification %**
				Grazing	Intensive feeding				Grazing	Intensive feeding	
X-residuals	1,5,1,1 no scatter	Real classification	Grazing	21	4	92.9%	Real classification	Grazing	4	3	73.3%
			Intensive feeding	0	31			Intensive feeding	1	7	
	2,5,1,1 no scatter		Grazing	22	3	89.3%		Grazing	7	0	100%
			Intensive feeding	3	28			Intensive feeding	0	8	
	1,5,1,1 SNV+DT		Grazing	24	1	98.2%		Grazing	7	0	100%
			Intensive feeding	0	31			Intensive feeding	0	8	
	2,5,1,1 SNV+DT		Grazing	25	0	98.2%		Grazing	7	0	93.3%
			Intensive feeding	1	30			Intensive feeding	1	7	
Maximum X-residuals		**Groups**		**Model prediction**		**Classification %**	**Groups**		**Model prediction**		**Classification %**
				Grazing	Intensive feeding				Grazing	Intensive feeding	
	1,5,1,1 no scatter	Real classification	Grazing	25	0	100%	Real classification	Grazing	5	2	86.7%
			Intensive feeding	0	31			Intensive feeding	0	8	
	2,5,1,1 no scatter		Grazing	24	1	96.4%		Grazing	6	1	93.3%

	Intensive feeding	1	30		Intensive feeding	0	8	
1,5,1,1 SNV+DT	Grazing	25	0	100%	Grazing	6	1	93.3%
	Intensive feeding	0	31		Intensive feeding	0	8	
2,5,1,1 SNV+DT	Grazing	24	1	98.2%	Grazing	5	2	80%
	Intensive feeding	0	31		Intensive feeding	1	7	

No scatter: no scatter effect correction performed; SNV + DT: Standard Normal Variate and De-trending transformation.
